# Distribution of Cardiometabolic Risk Factors in School-Aged Children with Excess Body Weight in the Al Ain City, United Arab Emirates: A Cross-Sectional Study

**DOI:** 10.3390/children8100884

**Published:** 2021-10-02

**Authors:** Akshaya Srikanth Bhagavathula, Sania Al-Hamad, Javed Yasin, Elhadi H. Aburawi

**Affiliations:** 1 Institute of Public Health, College of Medicine and Health Sciences, UAE University, Al Ain 17666, United Arab Emirates; 201890132@uaeu.ac.ae; 2 Department of Pediatrics, College of Medicine and Health Sciences, UAE University, Al Ain 17666, United Arab Emirates; saniaalhamad@uaeu.ac.ae; 3 Department of Medicine, College of Medicine and Health Sciences, UAE University, Al Ain 17666, United Arab Emirates; javed.yasin@uaeu.ac.ae

**Keywords:** body mass index, children, obesity, cardiometabolic, cardiovascular, United Arab Emirates

## Abstract

(1) Background: This study aimed to examine the distribution of cardiometabolic risk factors (CMRF) in school-aged children with excess body weight (overweight and obese) in Al Ain City, United Arab Emirates and identify the factors associated with increased cardiovascular risk factors between boys and girls. (2) Methods: A cross-sectional survey of children aged 6–17 years was conducted in Al Ain from 1 August 2019 to 31 December 2020. Binary logistic regression analysis was performed to investigate the relationship between excess body weight and CMRF between the groups and reported odds ratios (OR) with 95% confidence intervals (CI). (3) Results: A total of 966 school-aged children (490 boys and 476 girls) participated in the study, and the mean age of the children was 11.8 ± 2.9 years. The proportions of overweight and obesity were 13.5% and 10.2% in boys and 11.1% and 10.3% in girls. Higher glucose of ≥100 mg/dL (26.4%), triglycerides of ≥150 mg/dL and low-density lipoprotein cholesterol: ≥130 mg/dL (23.2%) were more prevalent in children with excess body weight. These children were at least two times more likely to have higher triglycerides levels, high total cholesterol (≥200 mg/dL) in girls (OR:2.06, 95% CI: 1.01–4.21) and low high-density lipoprotein (<35 mg/dL) in boys (OR: 2.20; 95% CI: 1.12–4.31). (4) Conclusions: Excess body weight in school-aged children was associated with increased CMRF, particularly triglycerides.

## 1. Introduction

The rising overweight and obesity epidemic among children in recent years has led to heightened awareness and concern about cardiovascular and metabolic health in school-aged children (typically 5–18 years) [1]. Previous studies have affirmed that the prevalence of cardiovascular risk factors in childhood has increased, and biomarkers of adverse cardiovascular outcomes have been found in children with obesity [2]. Severe obesity in children increases carotid artery wall stiffness and is associated with endothelial dysfunction (a marker of atherosclerosis) [3,4]. Recent studies have also reported that excess body weight (overweight and obesity) in children and adolescents has important implications for developing cardiovascular disease (CVD) and diabetes [5,6]. Moreover, cardiometabolic risk factors (CMRF) are more prevalent among children with excess weight than those of a healthy weight [7]. Two studies from the Emirates of Sharjah and Abu Dhabi, United Arab Emirates (UAE), have asserted that the prevalence of overweight and obesity in school-aged children (28.2%) and (29.7%), respectively, was four times higher (28.2%) than the global prevalence (6.7%) [8,9], while the prevalence rates of high blood pressure (BP) (≥95th percentile of centers for disease control criteria) were 15.4% among school-aged boys and 17.8% among girls [9]. 

In 2000, the Centers for Disease Control and Prevention (CDC) introduced the clinical use of the body mass index (BMI; in kg/m^2^) by sex- and age-specific growth charts for children and adolescents [10]. It is widely employed to screen children from two years of age onwards to identify those at risk for morbidity related to excess body fat [7,11,12,13,14,15]. Several medical societies have provided evidence-based guidelines for the primary prevention of atherosclerotic CVD beginning in childhood [16,17,18] and have defined CMRF specific to the age of children [19,20,21,22]. Nonetheless, minimal data are available regarding the distribution and association between excess body weight and CMRF in school-aged children in the Al Ain City, UAE. The prevalence of CMRF accompanying school-aged children was mostly heterogeneous due to the high proportion of expatriates (88.5%) living in the UAE. Thus, the findings from previous studies may not be representative of UAE nationals. Therefore, this study examined the distribution of CMRF in a representative sample (UAE nationals) of school-aged children and identified the factors associated with increased CVD risk factors among boys and girls with excess weight in the Al Ain City, UAE.

## 2. Materials and Methods

### 2.1. Study Design

A cross-sectional survey of the Al Ain City representative sample of Emirati School children aged 6–17 years was conducted between 1 August 2019 and 31 December 2020. 

### 2.2. Study Subjects

The study subjects comprised children who were aged 6 to 17 years at the time of examination, and a two-stage sampling method was used to select the schools and the students within each school. As schools are sex-specific, we stratified the schools to collect a similar number of boys and girls. Of 171 schools in Al Ain registered under the Abu Dhabi Education Council (ADEC), 34 schools were randomly selected (using random number table) as a first-stage sampling frame based on school size and the proportion of boys and girls enrolled in each school. In addition, the students were randomly selected using a systematic sampling technique through a computer-generated list of random numbers in the second-stage sampling by selecting even numbers of students according to the ADEC school list. We also sought a total sample size of 1600, expecting a 20–25% refusal rate, and then we increased the invited sample to 2000. A total of 2009 students and their parents signed the informed consent form. Of these, we excluded 216 participants who were underweight (<5th percentile) for age- and sex-specific BMI, as defined by CDC 2000 [10], and/or did not complete the clinical investigations (glucose, lipid parameters, and inflammatory markers) related to the study objectives (*n* = 827). Thus, the final sample was composed of 966 children (490 boys and 476 girls) from grades 2 to 10.

### 2.3. Anthropometric Measurements

All consenting students had anthropometric measurements taken (weight, height, and waist circumference) and were physically examined by a trained nurse. A stadiometer (a portable digital scale) was utilized to measure weight and height, and the children were asked to stand straight with their heads, backs, and buttocks vertically aligned to the height gauge. Thereafter, their heights were taken and rounded to the nearest 0.5 cm. Furthermore, waist circumference was measured using upstretched tapes, with the midpoint between the bottom of the rib cage and the tip of the iliac crest. The gender-specific body mass index BMI-for-age US growth charts was also used to identify normal weight (5th ≤ BMI < 85th percentile), overweight (BMI ≥85th percentile and <95th percentile), and obesity (BMI ≥ 95th percentile [23]. Neck circumference was measured below the laryngeal prominence and perpendicular to the long axis of the neck, and minimal circumference was recorded to the nearest 0.1 cm [24]. Body fat composition, body fat-free mass in kg, and body fat mass in percentage were also measured using a Tanita Body Composition Analyzer (TBF-300) by Tanita Corporation, Tokyo, Japan [25].

### 2.4. Clinical Variables

Non-fasting blood samples were collected by venipuncture in the early morning, and a reminder was sent to children’s parents the day before the blood was drawn. Blood samples (5–7 mL from each child) were also collected and kept in thermal boxes and transported to a laboratory within 2–3 h for analysis. Moreover, the following parameters were evaluated: blood glucose, hemoglobin A1c (HbA1c), total cholesterol (TC), low-density lipoprotein (LDL-C), high-density lipoprotein (HDL-C), triglycerides (TG), and gamma-glutamyl transferase (GGT) using an automated analyzer Integra 400 Plus (Roche Diagnostics, Mannheim, Germany). The enzyme-linked immunosorbent assays from R&D systems were also employed to measure adiponectin (Acrp30 Quantikine, DRP300), interleukin-6 (IL-6, using Human IL-6 Quantikine HS, HS600B), tumor necrosis factor-α (TNFα, Human TNFα Quantikine, DTA00C), soluble intercellular cytoadhesive molecule-1 (sICAM-1), soluble vascular cytoadhesive molecule-1 (sVCAM-1), and adiponectin (Acrp30 Quantikine, DRP300), following the manufacturers’ protocols. High-sensitivity C-reactive protein (hsCRP) was also measured using a Synchron Clinical System (UniCel DxC-800) from Beckman Coulter, Inc. (Fullerton, CA, USA). Further, the laboratory performed internal quality controls before running the samples and participated in the External Quality Assurance program through the College of American Pathologists Proficiency Testing.

Blood pressure (BP) was measured using a calibrated Omron M6 IntelliSense (Healthcare, Kyoto, Japan) automatic BP monitor, and the sleeves were suitable for each arm size. The measurements were also performed after the children had rested for five minutes in an air-conditioned environment, and three BP measurements were taken on the right arm with a five-minute interval between them. The average of these three measurements was registered.

### 2.5. Definition of Risk Factors

The definitions of normal body weight (5th ≤ BMI < 85th percentile), and excess body weight: overweight (BMI in the ≥85th and <95th percentiles), and obesity (BMI in the ≥95th percentile) were based on the recommendations of the CDC 2000 growth charts [10]. We utilized the standardized age-specific cut-off values to define abnormal values for TC: ≥200 mg/dL, HDL-C: <35 mg/dL, LDL-C: ≥130 mg/dL, TG: ≥150 mg/dL, systolic BP (SBP), and diastolic BP (DBP): ≥95th percentile or ≥130/80 mmHg, HbA1c: >5.7%), and glucose: ≥100 mg/dL) [7,16,19]. Table 1 presents the age for the sampling frame, the number of subjects, and the definitions used for abnormal values.

### 2.6. Statistical Analysis

All the estimates of precision are presented at a 95% confidence interval (CI) in the tables. The descriptive analysis included frequencies, percentages, and mean ± standard deviations (SD) where appropriate. The Pearson chi-square test or the Student *t*-test was also employed to evaluate the differences in the baseline characteristics between the boys and girls. The distribution of CMRF risk factors by age and gender in children with excess body weight was calculated and compared with the normal body weight category. The sex-specific odds ratios (OR) for the presence of risk factors in the participants with excess body weight were compared with the participants with normal body weight modeled in the binary logistic regression, which was adjusted for covariates. Logistic regression analyses were conducted separately for the boys and girls, and normal body weight was considered as a reference (OR,1) category. Statistical significance was considered if *p* < 0.05, and statistical analyses were performed with SPSS (Statistical Package for the Social Sciences) version 24.0 for Windows (SPSS, Chicago, IL, USA).

### 2.7. Ethical Considerations

The study protocol was approved by the Human Ethics Committee, College of Medicine and Health Sciences, United Arab Emirates University (AAMD-ERH_2019_6049), Al Ain, UAE on 4 June 2019. The purpose and importance of the study were explained to children’s parents, and written consent was obtained prior to their participation. Confidentiality was maintained by not disclosing personal information, and participants’ information was anonymized. 

## 3. Results

Among the 966 children, 50.7% were boys (mean age ± SD: 12.0 ± 2.9 years) and 74% were girls (11.6 ± 2.9 years). Overall, 218 (22.5%) children had excess body weight, 6.8% (*n* = 66) of the boys were overweight, and 5.1% (*n* = 49) of the girls were obese. No significant differences were found in the sociodemographic profile or in the prevalence of overweight and obesity according to sex. However, a higher proportion of girls are overweight (13.7%) and obese (12.1%) than boys (10.7% & 6.1%).

The mean values for cardiometabolic and inflammatory/endothelial function biomarkers were examined separately in boys and girls and are detailed in Table 2. The mean levels of pulse rate, waist circumference, body fat mass, LDL-C, HDL-C, TG, glucose, and gamma-glutamyl transferase (GGT) differed significantly by sex. Significant higher pulse rate (86.8 ± 11.8 beats/min) was observed in girls than boys (84.0 ± 12.9). In contrast, boys had significantly higher waist circumference (70.4 ± 17.3 cm), waist-to-height ratio (0.47 ± 0.10%), and body fat mass (46 ± 27.5 kg). The mean concentrations of LDL-C (101.0 ± 28.7 mg/dL) and HDL-C (52.3 ± 12.8 mg/dL) were significantly higher in girls, and the TG (100.5 ± 131.5 mg/dL) were significantly higher in boys than girls (90.4 ± 68.5 mg/dL). Moreover, boys also had significantly higher levels of glucose (91.6 ± 21.9 mg/dL) and GGT (24.2 ± 10.7). However, no significant differences in the inflammatory/endothelial function markers such as Interleukin 6 (*p* = 0.503), TNF alpha (*p* = 0.778), C-reactive protein (*p* = 0.192), ICAM (*p* = 0.355) and VCAM-1 (*p* = 0.470) in boys and girls. More details are available in Table 2. 

**Figure 1:**
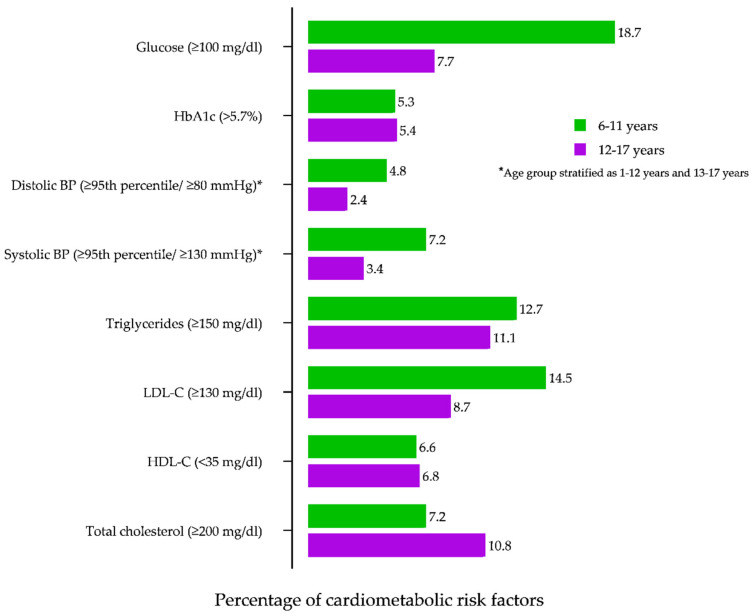
Distribution of cardiometabolic risk factors in children with excess body weight. HDL—high-density lipoprotein; LDL—low-density lipoprotein, BP—blood pressure.

Figure 1 depicts the distribution of each CMRF in children with excess body weight variable by age. Among the participants between 6 and 11 years of age, the prevalence of all the risk factors is higher, with the exception of TC among those 12 to 17 years of age, and higher glucose (≥100 mg/dL) in those aged < 12 years. Moreover, a higher proportion of TG and LDL-C levels were observed in the school children.

Table 3 shows the statistical significance of differences in cardiometabolic risk factors between excess body weight and normal body weight. Overall, low HDL-C (25.2%), and higher TG (14.2%), glucose (14.2%) and LDL-C (11.5%) are common in children with excess body weight. A significant higher TG and low HDL-C were apparent in boys than girls. 

Table 4 shows the results of the binary logistic regression models that controlled for covariates, such as age, education, and number of CVD risk factors. Children with excess body weight were at least two times more likely to have higher TG levels than same-sex participants with normal body weight. In addition, excess weight girls showed higher odds (OR: 6.4) for elevated SBP and TC levels (OR: 2.0) relative to the normal-weight girls. Boys with excess weight were 2.2 times more likely to have lower HDL-C levels.

## 4. Discussion

In this cross-sectional study on school-aged children from Al Ain, UAE, we examined the extent of CMRF and evaluated the factors associated with increased CVD risk in girls and boys with excess weight. CMRF, such as TC, LDL-C, TG and glucose were more prevalent in school-aged children, while boys had low HDL-C levels. Furthermore, excess weight in children was associated with major CMRF, with an increase in the odds ratio of TG levels by at least two times and lower HDL-C levels by 2.2 times in boys. The SBP at the 95th percentile was significantly higher odds in excess weight girls (OR: 6.4) than normal-weight girls.

The undesirable values of cardiometabolic variables in children with excess body weight suggest an increased susceptibility to the premature development of CVD in childhood and may have adverse health effects in adulthood. The distribution of CMRF differed across age groups, and there was a higher proportion of CMRF in the children aged 6–11 years than 12 years or older, except for TC in those aged > 11 years. Prior studies have validated that high CMRF occurs in school-aged children [26,27] and demonstrated that children with high body weight had an increased risk of atherogenic and CMRF [26,27,28]. Our findings of greater risks of abnormal lipid and glucose levels support risk stratification based on the current recommendations in children with excess body weight [29,30,31]. Similar to our present study, several studies have observed higher CMRF in children with excess body weight compared with normal-weight children [32,33,34,35]. These data reinforce the influence of excess weight on CMRF in school-aged girls.

The differences in CVD risk factors between the male and female participants in our study are notable, and excess weight is associated with a higher prevalence of abnormal lipid levels among the male participants than the female participants. Similarly, excess weight girls had abnormal glucose, TG and TC levels. It is believed that CMRF develops earlier in boys than in girls, and our findings differ from previous reports that have used only standard definitions and shown minimal differences between boys and girls [36,37,38]. Therefore, the same definitions of CVD risk factors in boys and girls with excess body weight may not determine equivalent risk and warrant further elucidation.

The early recognition of CMRF during childhood is crucial to prevent cardiovascular and metabolic changes in the future. In our study, reduced HDL-C was a common CMRF present in excess weight boys, which is also something Brzezinski et al. [39] and Ahmadi et al. [40] found in school-aged children and adolescents. Furthermore, in our study, excess weight boys and girls had increased CVD risk factors such as higher TG, low HDL-C in boys, and elevated SBP in girls compared to normal-weight children. The relationship between excess body fat and CMRF has been reported in the literature, and researchers have explained that the chronic inflammatory process is caused by excess fat accumulated explicitly in the abdominal cavity [41,42,43]. In our previous report [43], we demonstrated that dyslipidemia was adversely associated with endothelial dysfunction in school-aged children and, through this research, we identified that excess weight children had concomitant lipid profile disorders and particularly increased TG levels at early ages. Our study findings imply that abnormal weight gain must be identified early, the primary prevention of CVD in children be prioritized, and the risks for undesirable structural and metabolic changes must ultimately be reduced. Children with higher mean values of CMRF require more attention; as teenagers’ transition to young adulthood, the screening of CMRF in this age group is essential. Findings from the Global Burden of Disease study highlighted that nearly 268 million children and adolescents would become overweight and obese by 2025 [44]. Hence, effective interventions to combat excess fat in UAE children are highly necessary.

In Al Ain, as one of the largest UAE cities, abnormal levels of cardiometabolic variables in school-aged children with excess body weight are high, particularly in girls. Identifying abnormal weight gain early, prioritizing the primary prevention of CVD in children, and reducing risks for undesirable structural and metabolic changes are essential. Moreover, concerted efforts are urgently crucial to prevent the adverse consequences of excess body weight on future generations’ health and wellbeing. Further large, longitudinal, and multinational studies are required to clarify CMRF in the adolescent age group at least.

Our study has some limitations to consider. First, the data were collected in a cross-sectional design; thus, the duration of exposure to the risk factors could not be estimated. Second, although we used all the standard procedures to measure CMRF, all the measurements were taken during one visit. Accordingly, the distribution of CMRF may not necessarily reflect the “true” prevalence, and the association may not be causative. Third, the students were not fasting for their lipid profile and glucose. Fourth, we adjusted for all the potential risk factors in the logistic regression analysis, and there was a probability of us having residual confounders. Fifth, due to the effect of the COVID-19 pandemic, 41.2% lower participation was recorded than expected, which resulted in refusal and reduced sample size. Lastly, the study was conducted among school-aged children living in Al Ain; therefore, the findings cannot be generalized for all UAE school children.

## 5. Conclusions

The prevalence of CRMF such as higher TG, TC, and glucose levels is higher in Emirati school-aged children with excess body weight. This may be due to the consumption of unhealthy food, a sedentary lifestyle, and family history. Identifying abnormal weight gain early, prioritizing the primary prevention of CVD in children, and reducing risks for undesirable structural and metabolic changes are essential. Moreover, concerted efforts are urgently required to prevent the adverse consequences of excess body weight on future generations’ health and wellbeing. Further large, longitudinal, and multinational studies are required to clarify CMRF in the adolescent age group at least.

## Figures and Tables

**Table 1:** Definition of cardiometabolic risk factor variables.

Variable	Age (Years)	No. Of Participants Evaluated	Definition of Abnormal Values
Total cholesterol	6–17	966	≥200 mg/dL
HDL cholesterol	6–17	966	<35 mg/dL
LDL cholesterol	6–17	966	≥130 mg/dL
Triglycerides	6–17	966	≥150 mg/dL
Systolic BP	1–12	588	≥95th percentile
	13–17	378	≥130 mmHg
Diastolic BP	1–12	588	≥95th percentile
	13–17	378	≥80 mmHg
Glycated hemoglobin	11–17	811	>5.7%
Glucose	11–17	811	≥100 mg/dL

HDL: high-density lipoprotein; LDL: low-density lipoprotein; BP: blood pressure.

**Table 2:** Characteristics of the study population (*n* = 966).

Characteristics	Boys (*n* = 490, 50.7%)	Girls (*n* = 476, 49.3%)	*p*-Value *
**Age, %, 95% CI**			0.809
6–11 years	26.7 (22.8–30.9)	26.1 (22.1–30.2)	
12–17 years	73.2 (69.1–77.1)	73.9 (69.7–77.8)	
**Schooling**			0.127
2–5 grades	14.9 (11.9–18.3)	19.7 (16.2–23.6)	
6–9 grades	45.7 (41.2–50.2)	42 (37.5–46.6)	
10 or above grade	39.4 (35.0–43.8)	38.2 (33.8–42.7)	
**BMI category ^†^**			
**≤10 years old**			0.160
Overweight	10.7 (5.9–17.2)	13.7 (8.2–21.0)	
Obese	6.1 (2.6–11.6)	12.1 (6.9–19.1)	
**11–17 years old**			0.122
Overweight	14.5 (11.0–18.5)	10.2 (7.2–13.8)	
Obese	11.7 (8.5–15.4)	9.7 (6.7–13.2)	
**Clinical parameters (mean ± SD)**			
Pulse rate, beats/min	84.0 ± 12.9	86.8 ± 11.8	**<0.001**
Blood pressure, mmHg	113.2 ± 71.1	111.8 ± 72.1	0.293
Waist circumference, cm	70.4 ± 17.3	64.0 ± 13.2	**<0.001**
Waist/height ratio, %	0.47 ± 0.10	0.44 ± 0.09	**<0.001**
Neck circumference, cm	32.2 ± 6.5	31.0 ± 8.9	0.116
Body fat mass, kg	46.0 ± 27.5	36.4 ± 6.4	**<0.001**
Body fat mass, %	12.7 ± 11.2	14.5 ± 10.3	0.115
**Lipid profile, mg/dL**			
Total cholesterol	164.9 ± 43.4	153.7 ± 26.6	0.474
Low-density lipoprotein cholesterol	98.8 ± 23.6	101.0 ± 28.7	**0.001**
High-density lipoprotein cholesterol	50.2 ± 15.0	52.3 ±12.8	**<0.001**
Triglycerides	100.5 ± 131.5	90.4 ± 68.5	**0.017**
Apolipoprotein A, g/L	1.5 ± 2.6	1.5 ± 0.3	0.419
Apolipoprotein B, g/L	2.1 ± 8.42	0.81 ± 0.29	0.257
Lipoprotein (a), mg/dL	59.9 ± 48.1	49.0 ± 45.2	0.480
**Inflammatory/endothelial function biomarkers**			
Interleukin 6 (g/L)	4.1 ± 2.6	3.8 ± 2.1	0.503
Intercellular adhesion molecule 1 (ICMA) ng/mL	252.3 ± 88.2	263.5 ± 79.1	0.355
Vascular cell adhesion molecule 1 (VCAM-1), ng/mL	593.7 ± 142.3	586.7 ± 128.0	0.470
Tumor necrosis factor alpha (TNF alpha), g/mL	7.9 ± 2.4	5.8 ± 2.4	0.778
Adiponectin µg/ml	7.9 ± 4.0	7.2 ± 3.3	0.572
C-reactive protein, g/L	1.6 ± 2.4	1.6 ± 2.5	0.192
**Metabolic parameters**			
Glucose	91.6 ± 21.9	90.0 ± 20.4	**0.012**
Glycated hemoglobin (HbA1c), %	5.2 ± 0.4	5.1 ± 0.4	0.097
Gamma-glutamyl transferase measured in U/L	24.2 ± 10.7	20.0 ± 6.6	**<0.001**

* *p* for differences between sexes (* Student *t*-test for continuous variables), CI: confidence intervals, BMI: body mass index, CVD: cardiovascular disease. SD: standard deviation. ^†^ BMI: body mass index (overweight—BMI ≥ 85th and <95th age- and sex-specific percentile values, obese—BMI ≥ 95th age- and sex-specific percentile values) according to the Centers for Disease Control and Prevention 2000 growth charts.

**Table 3:** Proportion of children with cardiometabolic risk factors by their body weight category.

Risk Factor, % (95% CI)	Normal Weight (*n* = 748)	Excess Body Weight (*n* = 218)	*p*-Value
**Total cholesterol (≥200 mg/dL)**	*n* = 50 (6.6)	*n* = 22 (10.1)	0.499
Boys	3.2 (2.0–4.7)	4.1 (1.9–7.7)	
Girls	3.4 (2.2–5.0)	5.9 (3.2–9.9)	
**HDL cholesterol (<35 mg/dL)**	*n* = 38 (5)	*n* = 55 (25.2)	**0.039**
Boys	3.3 (2.1–4.9)	7.3 (4.2–11.6)	
Girls	1.7 (0.9–2.9)	1.8 (0.5–4.6)	
**LDL cholesterol (≥130 mg/dL)**	*n* = 66 (8.8)	*n* = 25 (11.5)	0.263
Boys	5.0 (3.6–6.9)	6.4 (3.5–10.5)	
Girls	4.9 (3.5–6.7)	5.0 (2.5–8.8)	
**Triglycerides (≥150 mg/dL)**	*n* = 47 (6.2)	*n* = 31 (14.2)	**<0.001**
Boys	3.3 (2.1–4.9)	7.3 (4.2–11.6)	
Girls	2.9 (1.8–4.4)	6.8 (3.9–11.0)	
**Systolic BP** Age: <13 years (≥95th percentile)	*n* = 15 (6.8)	*n* = 6 (2.8)	0.777
Boys	1.4 (0.7–2.6)	1.3 (0.2–3.9)	
Girls	0.5 (0.1–1.3)	1.3 (0.2–3.9)	
Age: 13–17 years (≥ 130 mmHg)	*n* = 6 (3.7)	*n* = 10 (6.2)	**0.015**
Boys	0.8 (0.3–1.7)	3.2 (1.3–6.5)	
Girls	0	2.7 (1.0–5.9)	
**Diastolic BP** Age: <13 years (≥95th percentile)	*n* = 15 (6.8)	*n* = 4 (1.9)	0.686
Boys	1.3 (0.6–2.4)	1.3 (0.2–3.9)	
Girls	0.6 (0.2–1.5)	0.4 (0.1–2.5)	
Age: 13–17 years (≥80 mmHg)	*n* = 6 (3.7)	*n* = 6 (2.7)	0.287
Boys	0.8 (0.3–1.7)	2.3 (0.7–5.2)	
Girls	0	0.4 (0.1–2.5)	
**Glycated hemoglobin (>5.7%)** Age: 11–17 years	*n* = 44 (5.8)	*n* = 11 (5)	0.536
Boys	3.3 (2.1–4.9)	3.6 (1.6–7.1)	
Girls	2.5 (1.5–3.9)	1.3 (0.2–3.9)	
**Glucose (≥100 mg/dL)** Age: 11–17 years	*n* = 98 (13.1)	*n* = 31 (14.2)	0.766
Boys	6.4 (4.7–8.4)	10 (6.4–14.8)	
Girls	6.6 (5.0–8.7)	4.1 (1.9–7.7)	

*p* for differences across the BMI categories using χ2 statistics.

**Table 4:** Odds ratios for cardiovascular risk factors in children with excess body weight.

Variable	Boys		Girls	
	OR (95% CI)	*p*-Value	OR (95% CI)	*p*-Value
**Total cholesterol (≥200 mg/dL)**				
Normal weight	1	–	1	–
Excess weight	1.24 (0.56–2.77)	0.590	**2.06 (1.01–4.21)**	**0.046**
**HDL cholesterol (<35 mg/dL)**				
Normal weight	1	–	1	–
Excess weight	**2.20 (1.12–4.31)**	**0.021**	1.11 (0.35–3.51)	0.854
**LDL cholesterol (≥130 mg/dL)**				
Normal weight	1	–	1	–
Excess weight	1.58 (0.80–3.11)	0.186	1.11 (0.54–2.27)	0.767
**Triglycerides (≥150 mg/dL)**				
Normal weight	1	–	1	–
Excess weight	**2.08 (1.06–4.10)**	**0.033**	**2.74 (1.36–5.52)**	**0.005**
**Systolic BP (≥95th percentile)**				
Normal weight	1	–	1	–
Excess weight	1.39 (0.55–3.52)	0.485	**6.45 (1.83–22.72)**	**0.004**
**Diastolic BP (≥95th percentile)**				
Normal weight	1	–	1	–
Excess weight	1..23 (0.46–3.26)	0.673	1.73 (0.38–7.77)	0.472
**Glycated hemoglobin (>5.7%)**				
Normal weight	1	–	1	–
Excess weight	0.83 (0.37–1.86)	0.655	0.67 (0.22–2.01)	0.478
**Glucose (≥100 mg/dL)**				
Normal weight	1	–	1	-
Excess weight	1.30 (0.78–2.17)	0.313	0.83 (0.46–1.49)	0.545

OR: odds ratio; CI: confidence interval. Logistic regression adjusted for age, education, and number of CVD risk factors.

## Data Availability

The data that support the findings of this study are available on request from the corresponding author (EHA).
